# Forceps in Labor

**Published:** 1886-09

**Authors:** C. L. Gwyn

**Affiliations:** Galveston


### FORCEPS IN LABOR.

*By C. L. Gwyn, M. D.*

[Read before the Galveston Medical Club, July 5, 1886.]

[For Daniel's Texas Medical Journal.]

I WILL not tire you with an account of the first use and introduction as well as the various modifications of these invaluable instrument; their history is supplied by your text-books.

While valuing them highly as a sometimes indispensable aid in our troubles, it is their too frequent use, or their abuse, that gives me concern.

I will not be uncharitable enough to say that the younger members of our profession are impelled to the indiscriminate use of the forceps by their zeal, or goaded on by the clamor and importunities of the friends and attendants of the patients to have something done for speedy relief; or again that they are actuated by a desire to show their superior skill; nor will I accuse our seniors, the busy practitioner, of a desire to get rid of his case and save time, but in their needless use they will find that a Nemesis will follow them.

I say these things with a full knowledge that I will be voted non-progressive and an "old fogy," but Gentlemen, I would rather be considered a "slow coach", "not up to the times," than needlessly or carelessly mutilate a single woman; I care not how low her condition may be in the scale of social life.

I will not repeat the rules for the use of these instruments, they are so plainly and well given in every well ordered work on Obstetrics, that if heeded, will prevent their abuse. Labor is a natural, a physiological condition, one for which the system is gradually prepared from the beginning of pregnancy, and any medicine, or the use of any instrument that interferes with this condition renders it pathological. This state of affairs is best illustrated by abortion, either from want of viability of the embryo, or brought on by induction; in either case we find the uterus unprepared for the change, and sub-involution a result.

Sub-involution with its chain of morbid symptoms is not the only ill we have to expect from the ill-advised use of instruments; we may, and frequently do have lacerations of cervix uteri, and of perineum or sometimes both. I do not claim that all cases of laceration are due to the use of forceps, but the majority are, and the poor woman a cripple for life, or until restored by the gynæic surgeons' act. I will hardly be amiss when I claim that the forceps make more work for the gynæcologist than all other conditions combined.

During the past year I have had in my practice seven or eight cases to present themselves for uterine troubles due to laceration. In six cases I found lacerations of the perineum, and in one case both of cervix and perineum, in which instruments had been used in delivery; also one case of laceration of perineum occurring naturally. Three of the cases claimed to have occurred in Hospital, three claimed to have suffered at the hands of resident physicians of this place, and one from the ignorance of a widwife. It may be objected that those occuring in Hospital were at the hands of resident students, tyros, yet they seem to do no worse than the older members of the craft.

It will, doubtless, be claimed that the use of instruments, by shortening labor, lessens the suffering and saves the strength of the woman, and that she has a good "getting up." Yet such is really not the case; for involution is interfered with, the natural, persistent and violent muscular contractions of the uterus hasten structural changes, fatty degeneration of the now useless tissues takes place more speedily, and the effete products are more readily thrown off as lochia, than if meddled with.

Dame Nature does her work well; art has never been able to equal, much less improve upon her methods, and we, as physicians, find that our greatest success in treatment of all disease is when we approach nearest to Nature's ways.

Our right to interfere with Nature's course in delivery for the relief of pain is questionable; we need go but a little further back and produce abortion, or prevent conception, if the ablation of pain is the great desideratum; the one is as justifiable as the other; the mental as well as the physical sequences of unfertile copulation, and the disasters that follow in the wake of abortion, would, if no other or moral reason, restrain the honorable physician from such unholy procedures.

In cases of impaction or apparent impaction ot the fœtal head, frequently external manipulations will facilitate labor by a slight change of the cranial position; and most of you have found that after introducing one blade of the forceps, the head commenced to descend and normal labor resumed.

The temptation to use forceps is very great in cases of inertia uteri, yet it has been my good fortune to be able to do without them more than once, by the exhibition of ergot, (a bad practice); by the use of stimulants, and by external manipulations.

I remember a case of inertia uteri in which the patient had general anasarca from malarial troubles, and probably nephritic complications, and was as marked a case of anæmia as one could wish to see. She had an intolerance of alcoholic stimulants; ergot failed to produce contractions; my patient was rapidly sinking from exhaustion; my forceps were six miles away, at home. I used inhalations of nitrite of amyl; as the cheek flushed the brain became nourished and the uterus contracted. Three exhibitions of the medicine, within a half hour, caused labor to continue, and a full term, living child to be brought into the world before the messenger returned with my instruments. In this case the amount of hemorrhage, following the birth of the child, was less than I have ever before had in a case of labor. It was one of those cases in which the use of forceps would have been justified; but what would have been the condition of my patient? I would probably have had a flabby, flacid and non-contracted uterus with every sinus open, and oozing the life-blood of my patient away, and, in all probability, she would have succumbed to an uncontrollable post-partum hemorrhage.

In still another, but opposite condition of affairs, the temptation is equally great. In the stout little woman, in full health, in the prime of her womanhood, when every muscle is toned up to its fullest extent and is rigid as a bar of iron, labor is often delayed by the non-yielding of the muscles of the external outlet. Relaxants will save your interference.

And, gentlemen, in conclusion, I feel as if I had but half done my duty to the profession and to humanity should I fail to lift up my voice and cry aloud from the watch-towers against the abuse and misuse of these potent engines of destruction which should be the "dernier ressort" of the obstetrician.

